# Exogenous Ca^2+^ priming can improve peanut photosynthetic carbon fixation and pod yield under early sowing scenarios in the field

**DOI:** 10.3389/fpls.2022.1004721

**Published:** 2022-09-29

**Authors:** Qiaobo Song, Siwei Zhang, Chunming Bai, Qingwen Shi, Di Wu, Yifei Liu, Xiaori Han, Tianlai Li, Jean Wan Hong Yong

**Affiliations:** ^1^ College of Land and Environment, National Engineering Research Center for Efficient Utilization of Soil and Fertilizer Resources, Northeast China Plant Nutrition and Fertilization Scientific Observation and Research Center for Ministry of Agriculture and Rural Affairs, Key Laboratory of Protected Horticulture of Education Ministry and Liaoning Province, Shenyang Agricultural University, Shenyang, China; ^2^ Research Institute of Sorghum, Liaoning Academy of Agricultural Sciences, Shenyang, China; ^3^ The UWA Institute of Agriculture, The University of Western Australia, Perth, WA, Australia; ^4^ School of Biological Sciences, The University of Western Australia, Perth, WA, Australia; ^5^ School of Agriculture and Environment, The University of Western Australia, Perth, WA, Australia; ^6^ Department of Biosystems and Technology, Swedish University of Agricultural Sciences, Alnarp, Sweden

**Keywords:** early sowing, peanut (*Arachia hypogeae*), cold tolerance, Ca^2+^, photosynthesis

## Abstract

Harnessing cold-resilient and calcium-enriched peanut production technology are crucial for high-yielding peanut cultivation in high-latitude areas. However, there is limited field data about how exogenous calcium (Ca^2+^) application would improve peanut growth resilience during exposure to chilling stress at early sowing (ES). To help address this problem, a two-year field study was conducted to assess the effects of exogenous foliar Ca^2+^ application on photosynthetic carbon fixation and pod yield in peanuts under different sowing scenarios. We measured plant growth indexes, leaf photosynthetic gas exchange, photosystems activities, and yield in peanuts. It was indicated that ES chilling stress at the peanut seedling stage led to the reduction of Pn, g_s_, Tr, Ls, WUE, respectively, and the excessive accumulation of non-structural carbohydrates in leaves, which eventually induced a chilling-dependent feedback inhibition of photosynthesis due mainly to weaken growth/sink demand. While exogenous Ca^2+^ foliar application improved the export of nonstructural carbohydrates, and photosynthetic capacity, meanwhile activated cyclic electron flow, thereby enhancing growth and biomass accumulation in peanut seedlings undergoing ES chilling stress. Furthermore, ES combined with exogenous Ca^2+^ application can significantly enhance plant chilling resistance and peanut yield ultimately in the field. In summary, the above results demonstrated that exogenous foliar Ca^2+^ application restored the ES-linked feedback inhibition of photosynthesis, enhancing the growth/sink demand and the yield of peanuts.

## 1 Introduction

Peanut (*Arachis hypogaea* L.) is an important oil and industrial crop that originated in tropical South America (Bolivia and adjacent countries), which is sensitive to chilling stress due to a lack of naturally-endowed cold acclimation (Prasad et al., 2006; Song et al., 2020; Liu et al., 2022). Considering the achievable net economic return, early sowing is a useful agricultural cultivation strategy to avoid drought stress during spring sowing and especially during the vulnerable seedling stages (Chen et al., 2012). Thus, it is paramount for peanut growers and researchers to uncover suitable sowing windows and cultivation durations in high-latitude areas to deliver successful peanut production (Dai et al., 2017; Li et al., 2018; Virk et al., 2020; Zhang et al., 2020). For the cropping boundary in the high-latitude crop, especially in response to low-temperature stress, the peanut’s light interception and strong sink demand are the major determinants of high yield and quality (Bagnall et al., 1988; Itoh, 2003; Salessesmith et al., 2020; Ensminger et al., 2006). However, the ES (early sowing) window in the most high-latitude area would naturally subject the crop to a greater probability of chilling exposure. Past studies have documented that chilling stress leads to a decrease in sink demand and lowers the capacity to utilize energy metabolically (Wan, 2003; Liu et al., 2013; Wu et al., 2020). The resultant is an imbalance source-to-sink relationship situation within the whole plant which affects many biochemical and physiological processes thereby curtailing growth. With more unpredictable and extreme weather events especially in high-latitude areas, establishing a simple exogenous chemical priming strategy to improve the cold adaptability of peanuts, is a major contribution toward improving peanut production (Song et al., 2020; Wu et al., 2020; Aryal and Sollenberger, 2021; Liu et al., 2022).

For optimizing the cropping season, adjusting the sown date and the harvest date are the fundamental prerequisites to guarantee a consistent and acceptable yield of peanuts (Chen et al., 2012; Dai et al., 2017; Zhang et al., 2020). In Northern China, early sowing, i.e. sowing around half a month earlier than current local agronomic practices, is widely used to avoid drought stress and maximize the yield of the new high-yielding big-seed type peanut cultivars in recent years (Wan, 2003; Zhang et al., 2017; Zhang et al., 2022; Liu et al., 2022). Past research has shown that 12°C and below are unfavorable cultivation temperatures for peanut physiology and growth. These low temperatures are common during the early spring/early sowing peanut cultivation scenario, especially low nocturnal temperature stress during the early spring night (Melkonian et al., 2004; Wu et al., 2020; Zhang et al., 2020). Previous studies have shown that chilling stress induced the peroxidation of membrane lipids (Zhang et al., 2000) and the accumulation of soluble sugars (Song et al., 2020; Wu et al., 2020), reduced stomatal conductance (Melkonian et al., 2004), reduced photosynthetic carbon fixation and the reaction centers’ activities (Long et al., 1983; Stirling et al., 2006; Friese and Sage, 2016; Yamori and Shikanai, 2016), and decreased activities of carbon assimilation enzymes (Riva-Roveda et al., 2016). Positively, several studies reported that appropriate early planting and late-season harvest produced adequate photosynthates and higher yields (Kasai, 2008; Srivastava et al., 2016). However, the apparent benefits of late-season harvest have to be evaluated with the higher risk of diseases; mold production (*Aspergillus flavus*) and pod loss, leaf spot disease, or even threatened by deadly extreme autumn chilling injury due to climate change (Fountain et al., 2015; Zhao et al., 2019).

Chemical priming is a promising measure in plant stress physiology and crop stress management (Nayyar, 2003; Fernández and Eichert, 2009). Exogenous foliar calcium (Ca^2+^) is absorbed by leaves through the hydrophilic pores in leaf stomata or epidermis which are affected by leaf temperature, relative humidity, light, and other factors (Li et al., 2020). In response to different chilling stresses, exogenous Ca^2+^ priming significantly improved the physiological response including growth and photosynthesis in low-temperature sensitive plant species such as peanuts (Liu et al., 2013; Zhang et al., 2017; Zhang et al., 2022; Liu et al., 2022), wheat (Nayyar, 2003), loquat fruit (Li et al., 2020), and tomato (Zhang et al., 2014). It is generally believed that plant cell walls, mitochondria, and chloroplasts have an enormous capacity to store Ca^2+^ (Tian et al., 2019). And optimal levels of Ca^2+^ can sustain cell wall growth and membrane integrity as well as osmotic functioning (Kreimer et al., 1988; Rocha and Vothknecht, 2012). The concentration of free Ca^2+^ in cytosol further improved the resilience to cold injury of plants (Kreimer et al., 1988; Brauer et al., 1990; Gan et al., 2019). Ca^2+^ is a cofactor of the oxygen-evolving complex (OEC), which is involved in light-induced water photodissociation (Thor, 2019), regulated the formation of assimilatory power (Wang et al., 2019) and the activity of carbon assimilation-related enzymes (Rocha and Vothknecht, 2012). Ca^2+^ is involved in regulating carbohydrate metabolism, including sucrose synthesis, which is implicated in phloem function (Rufty and Huber, 1983; Brauer et al., 1990; Tian et al., 2019; Kinose et al., 2020). Furthermore, the Ca^2+^/calmodulin interaction is involved in regulating NAD kinase, photosynthesis, and photoprotection (Klughammer and Schreiber, 1994; Liu, 2020). In particular, exogenous foliar Ca^2+^ application maintained peanut photosynthesis and growth during nocturnal chilling stress (Liu et al., 2013; Song et al., 2020; Wu et al., 2020; Liu et al., 2022). However, the underlying mechanisms of how exogenous foliar Ca^2+^ application enhanced peanut chilling resilience, growth, and yield is unclear under early sowing-induced moderate/natural fluctuating chilling stress (field conditions). Therefore, this study examined the effects of exogenous foliar Ca^2+^ application on seedling growth, leaf photosynthetic reactions, nonstructural carbohydrates accumulations, and yield under early sowing scenarios in the field. Those mentioned above are vital to improving and stabilizing peanut production in Northern China facing more unpredictable and extreme weather events.

## 2 Materials and methods

### 2.1 Plant material and experimental design

**Figure 1:**
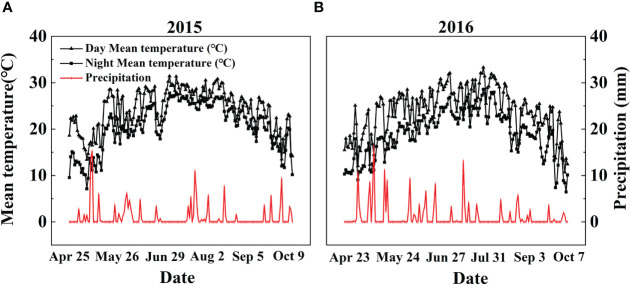
Daily precipitation and day/night mean temperatures during the **(A)** 2015 and **(B)** 2016 peanut-growing seasons in Shenyang, Northeast China.

A two-year field experiment was conducted at a farm station of Liaoning Academy of Agricultural Sciences, China (41°82’ N, 123°56’ E) in 2015 and 2016. The study area has a temperate monsoon climate with an average annual air temperature of 12.1°C. The photoperiod (daylight + twilight) varied within the range of 11-15 hr in the natural field of Liaoning province. The average annual rainfall is 604.8 mm, with the main precipitation season from Jun. to Sep. The Fenghua No.1 (abbreviated as FH1), the common high-yielding big-seed peanut cultivar (Shi et al., 2020), was used in this study. The farm soil was a simply cultivated wet-leached soil developed from Quaternary loess parent material with pH 6.56 (1:2.5, w/v), 12.86 g kg^-1^ organic matter, 1.36 g kg^-1^ total N, 48.52 mg kg^-1^ Olsen-P, 64.29 mg kg^-1^ available K, 351.92 mg kg^-1^ exchangeable Ca, 12.7 cmol kg^-1^ cation exchange capacity, and 1.49 g cm^-3^ bulk density. Daily meteorological data, including the average day and night air temperature and daily precipitation, were recorded by an automatic meteorological station located ~60 m from the experimental site. The average day and night air temperature (°C) and daily precipitation (mm) from early sowing on Apr. 25, 2015, and Apr. 23, 2016, to late harvest on Oct. 9, 2015, and Oct. 7, 2016, were recorded, respectively (**Figure 1**).

The field experiments were conducted from Apr.25 to Oct.9 of 2015 and from Apr.23 to Oct.7 of 2016. The experimental design involved a randomized 12 plots with four treatments and three replicates of each treatment (3 plots/replicates per treatment). The plot size was 4 m long and 3 m wide with three beds per plot, and each bed contained two rows of peanuts which were planted at 0.2 m intervals, with two plants per hill. The number of peanut seedlings planted in each plot was calculated as follows: No. = (4 m/0.2 m) × 3 (beds/plot) × 2 (rows/bed) × 2 (peanuts/row) = 240 seedlings/plot. Peanut special biochar-based fertilizer (N-P_2_O_5_-K_2_O=13-15-17, Liaoning Jinhefu Agricultural Science and Technology Co. Ltd, China) was applied and mixed evenly in the plough layer (depth of approximately 20 cm) to each plot at the time of planting with 750 kg ha^-1^. The pre-sowing herbicide, trifluralin (α,α,α-trifluoro-2,6-dinitro-N, N-dipropyl-p-toluidine), was applied to the soil, and the plots were kept weed-free thereafter by hand weeding. Other planting inputs were consistent with current local agronomic practices. The treatments were (1) CK (Normal sowing according to the local farmer’s preference and current agronomic practices + foliar spraying of ddH_2_O); (2) CK+Ca (Normal sowing + foliar spraying of 15 mM CaCl_2_); (3) ES (Early sowing + foliar spraying of ddH_2_O); (4) ES+Ca (Early sowing + foliar spraying of 15 mM CaCl_2_). The optimal levels of exogenous foliar Ca^2+^ (15 mM CaCl_2_) and the application techniques (the leaves were sprayed evenly until just dripping) were established in the previous relative peanut studies (Liu et al., 2013; Song et al., 2020; Wu et al., 2020; Liu et al., 2022). As for early sowing treatments (ES, ES+Ca), peanuts sowing dates were on April. 25, 2015, and April. 23, 2016, respectively. While as for normal sowing treatments (CK, CK+Ca), peanuts’ sowing dates were on May. 9, 2015, and May. 7, 2016, respectively. At the peanuts seedling stages (≥3 leaves), all plants were foliar-sprayed carefully and evenly using moisture sprayers twice a week (every Wednesday and Friday from May. 20 to Jun. 5 of 2015, and May. 18 to Jun. 3 of 2016) plus twice a day (at around 8:30 am and 4:30 pm) with 15 mM CaCl_2_ (CK+Ca and ES+Ca) or ddH_2_O (CK and ES).

### 2.2 Plant sampling and measurements

#### 2.2.1 Plant morphological indexes and nonstructural carbohydrates measurements

All plant sampling and measurements were carried out every 7 days at the seedling stage from May. 23, 2015, and May. 21, 2016 onward, respectively. The main stem height was estimated carefully using a meter scale. The leaf area was measured using LI-3000C (LI-COR Biosciences, Lincoln NE, USA). All samples were oven-dried at 105°C for 0.5 hr and then at 70°C to a constant weight. Also, oven-dried leaflets from the third youngest fully expanded leaves were pooled by treatment and ground to a powder, and triplicate subsamples were used for carbohydrate measurements. Soluble sugars were extracted from approximately 0.1 g of the oven-dried leaf powder with 80% (v/v) ethanol at 85°C and quantified using the microtiter method (Hendrix, 1993). Pellets containing starch were oven-dried overnight at 60°C. Starch in the pellet was first gelatinized by the addition of 1 ml of 0.2 M KOH and incubated in a boiling water bath for 0.5 hr (Rufty and Huber, 1983). After cooling, 0.2 ml of 1 M acetic acid was added, and the solution was incubated with 2 ml acetate buffer (pH 4.6) containing amyloglucosidase (6 units, Roche) at 55°C for 1 hr. The reaction was terminated in a boiling water bath, and the resulting supernatant was analyzed for glucose (Song et al., 2020; Wu et al., 2020; Liu et al., 2022).

#### 2.2.2 Peanut yield measurement

Peanut yield was measured at different harvesting times [i.e. Sep. 25 (early harvest) and Oct. 9 (late harvest), 2015 or Sep. 23 (early harvest) and Oct.7 (late harvest), 2016]. During the early harvest in September, the reproductive period of the CK is 139 d, and the ES is 153 d. During the late harvest in October, the CK is 153 d, and the ES is 167 d. In order to improve the peanut yield estimation and accuracy in each plot, samples were collected in the specific one-third area of the experimental plot; when harvested at different times, the other two-thirds area of the plot was harvested to calculate the pod yield (Kramer et al., 2004). The pod yield was determined on Sep.25 (early harvest), Oct.9 (late harvest), 2015, and Sep.23 (early harvest), Oct.7 (late harvest), 2016, respectively. For representative sampling, an area of 2 × 2 m^2^ in the center of each plot was selected accordingly. Plants were harvested at maturity (100 days after planting) and the number and dry matter per pot were determined. These pods were air-dried for 1 week and then weighted.

#### 2.2.3 Leaf gas exchange, chlorophyll fluorescence and P700 measurements

On each observation date, the chlorophyll fluorescence was measured at 6:00 am after a dark adaptation of 1 hr, and leaf gas exchange was measured at 10:00 am. Leaf gas exchange was measured on the third youngest fully expanded leaves using an open system of gas exchange equipment (GFS-3000, Heinz Walz GmbH, Effeltrich, Germany). During gas exchange measurements, an LED array provided a PPFD 1000 μmol·quanta·m^-2^·s^-1^, and the leaf cuvette temperature was set to 25°C and 60% RH. The CO_2_ concentration was maintained at 400 ± 5 μmol·mol^-1^. The third youngest fully expanded leaf was kept in the chamber by ensuring the thermo-couple touching it from the underside. Gas exchange parameters included net photosynthetic rate (Pn), stomatal conductance (g_s_), atmospheric CO_2_ concentration (Ca), transpiration rate (Tr), intercellular CO_2_ concentration (Ci), water-use efficiency (WUE), and leaf stomatal limitation (Ls).

The chlorophyll fluorescence and P700 parameters on the third youngest fully expanded leaf (*ca.* 1 cm^2^) were made using the Dual-PAM 100 measuring system (Heinz Walz, Effeltrich, Germany) which is controlled by Dual-PAM V1.19 software. All steps were carried out following the standard protocols and appropriate modifications (Shi et al., 2020; Song et al., 2020; Wu et al., 2020; Liu et al., 2022). The fluorescence slow-kinetics were measured after a dark adaptation of 1 hr, and the intensity of saturation pulse light (red light) and actinic light (red light) were set as 10,000 and 132 μmol·quanta·m^-2^·s^-1^, respectively. The chlorophyll fluorescence parameters were calculated by Fo, Fm, Fo’, Fm’, F. The Fo and Fm are respectively the minimum and maximum fluorescence yield of the dark-adjusted sample with the PSII center open and closed, while the Fo’ and Fm’ are the illuminated sample with some PSII center open and closed. The maximal/intrinsic photochemical efficiency of PSII (Fv/Fm) is calculated by  Fv/Fm=(Fm−Fo)/Fm (Klughammer and Schreiber, 1994). The actual quantum yield of PSII [Y(II)] is calculated by Y(II)=(Fm"−F)/Fm (Genty et al., 1989). The non-regulated energy loss in PSII [Y(NO)] is calculated by  Y(NO)=F/Fm (Klughammer and Schreiber, 2008). The regulatory quantum yield in PSII [Y(NPQ)] is calculated by Y(NPQ)=1−Y(II)−Y(NO) (Kramer et al., 2004). The relative electron transfer rate in PSII [ETR(II)] is calculated by ETR(II)=PAR·Y(II)·0.84·0.5 is photosynthetically active radiation which is 184 μmol·quanta·m^-2^·s^-1^ in this study (Yamori and Shikanai, 2016).

The PSI parameters were based on the redox kinetics of P700 induced by far-red light (ΔA830-ΔA875, P700^+^), as well as the PSII was measured by the Dual-pam 100 device and based on the previous studies (Shi et al., 2020; Song et al., 2020; Wu et al., 2020; Liu et al., 2022). The P700 parameters were calculated as: The actual quantum yield in PSI [Y(I)] is calculated by Y(I)=(Pm"−P)/Pm . The quantum yield of PSI non-photochemical energy dissipation due to the donor-side limitation [Y(ND)] is calculated by Y(ND)=P/Pm . The quantum yield of PSI non-photochemical energy dissipation due to the acceptor-side limitation [Y(NA)] is calculated by Y(NA)=(Pm"−P)/Pm . The cyclic electron flow (CEF) was estimated as CEF=ETR(I)−ETR(II) . Similarly, Y(CEF)=Y(I)−Y(II) was calculated as the ratio of the quantum yield of CEF to Y(II) and later used to estimate cyclic electron transfer.

### 2.4 Statistical analyses

Statistical analyses were carried out using one-way ANOVA in SPSS 19.0 (Chicago, IL, USA). Values are means ± SD (n= 3). Different letters indicate significant differences according to Duncan’s multiple range tests (*p* < 0.05). All graphs were plotted using Origin 8.0 and Excel 2016 software.

## 3 Results

### 3.1 Effects of exogenous foliar calcium (Ca^2+^) application on peanut growth under early and normal sowing scenarios

**Figure 2:**
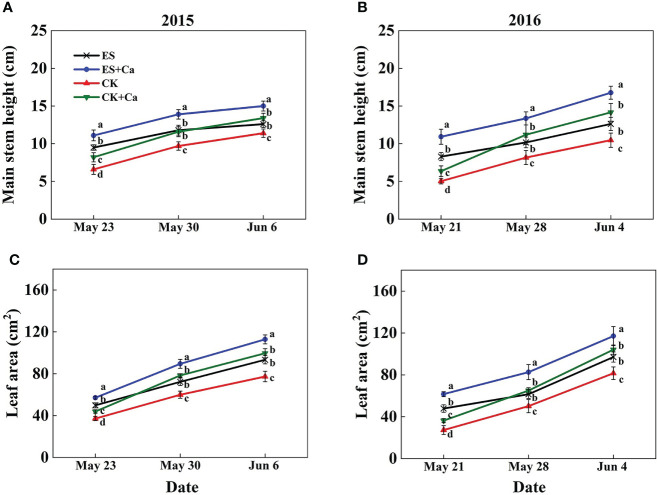
Effects of exogenous foliar calcium (Ca^2+^) application on peanut main stem height **(A, B)** and leaf area **(C, D)** under early and normal sowing scenarios. Values are means ± SD (n = 3). Different letters indicate significant differences according to Duncan’s multiple range tests (*p* < 0.05).

The ES treatment significantly enhanced the main stem height (10.5% in Jun. 6, 2015 and 20.6% in Jun. 4, 2016) and leaf area (20.8% in Jun. 6, 2015 and 19.1% in Jun. 4, 2016) compared with that of the CK. Meanwhile, ES+Ca and CK+Ca treatments further increased main stem height (ES+Ca: 19.0%, CK+Ca: 6.3% in Jun. 6, 2015) and leaf area (ES+Ca: 20.8%, CK+Ca: 6.6% in Jun. 6, 2015) than that of ES treatment (seedling stage, **Figure 2**).

**Figure 3:**
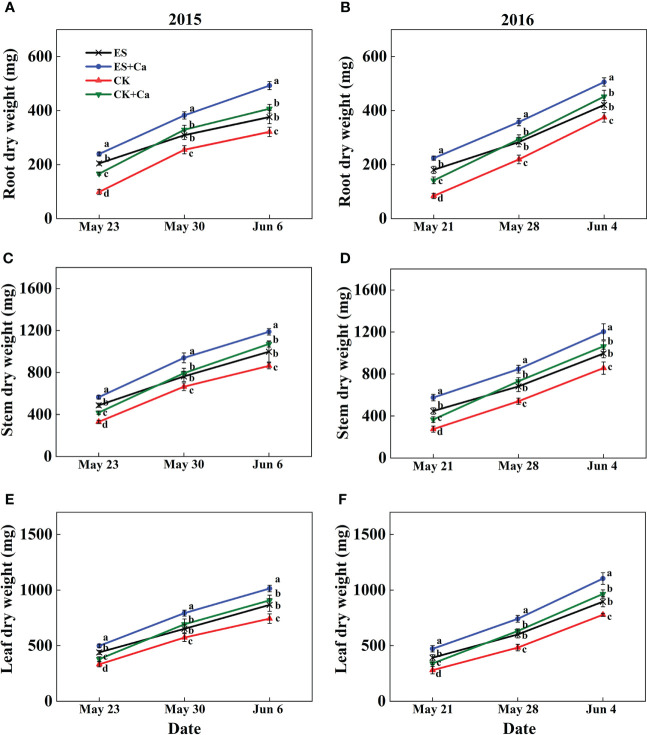
Effects of exogenous foliar calcium (Ca^2+^) application on peanut root dry weight **(A, B)**, stem dry weight **(C, D)**, and leaf dry weight **(E, F)** under early and normal sowing scenarios. Values are means ± SD (n = 3). Different letters indicate significant differences according to Duncan’s multiple range tests (*p* < 0.05).

The ES treatment enhanced dry matter accumulation in roots (17.1% in Jun. 6, 2015 and 12.2% in Jun. 4, 2016), stems (15.6% in Jun. 6, 2015 and 16.2% in Jun. 4, 2016), and leaves (16.6% in Jun. 6, 2015 and 15.1% in Jun. 4, 2016) compared with that of CK. Meanwhile, ES+Ca and CK+Ca treatments further enhanced dry matter accumulation in roots (ES+Ca: 30.8%, CK+Ca: 8.2% in Jun. 6, 2015), stems (ES+Ca: 19.0%, CK+Ca: 7.4% in Jun. 6, 2015), and leaves (ES+Ca: 17.0%, CK+Ca: 4.7% in Jun. 6, 2015, seedling stage, **Figure 3**).

### 3.2 Effects of exogenous foliar calcium (Ca^2+^) application on soluble sugar, starch, and total nonstructural carbohydrates concentrations of peanut leaves under early and normal sowing scenarios

**Figure 4:**
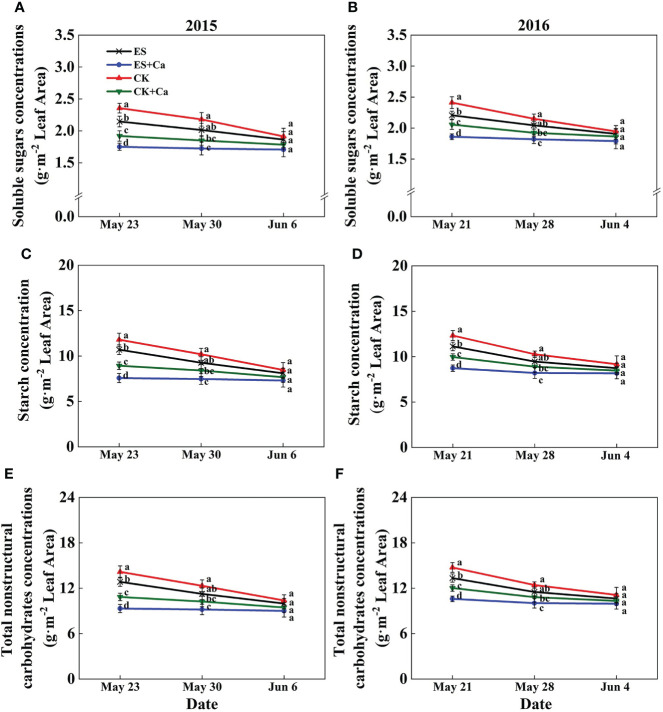
Effects of exogenous foliar calcium (Ca^2+^) application on peanut leaf soluble sugars concentrations **(A, B)**, starch concentration **(C, D)**, and total nonstructural carbohydrates concentrations **(E, F)** under early and normal sowing scenarios. Values are means ± SD (n = 3). Different letters indicate significant differences according to Duncan’s multiple range tests (*p* < 0.05).

The ES treatment reduced soluble sugar (2.6% in Jun. 6, 2015 and 2.1% in Jun. 4, 2016), starch (4.4% in Jun. 6, 2015 and 4.8% in Jun. 4, 2016), and total nonstructural carbohydrates (4.0% in Jun. 6, 2015 and 4.2% in Jun. 4, 2016) concentrations compared with that of CK. Meanwhile, ES+Ca and CK+Ca treatments further decreased soluble sugar (ES+Ca: 8.1%, CK+Ca: 4.3% in Jun. 6, 2015), starch (ES+Ca: 9.9%, CK+Ca: 5.3% in Jun. 6, 2015), and total nonstructural carbohydrates (ES+Ca: 9.5%, CK+Ca: 5.1% in Jun. 6, 2015) concentrations than that of ES treatment. However, the levels of soluble sugar, starch, and total nonstructural carbohydrates had no significant difference between all treatments on Jun. 6, 2015, and Jun. 4, 2016 (**Figure 4**).

### 3.3 Effects of exogenous foliar calcium (Ca^2+^) application on the gas exchange under early and normal sowing scenarios

**Figure 5:**
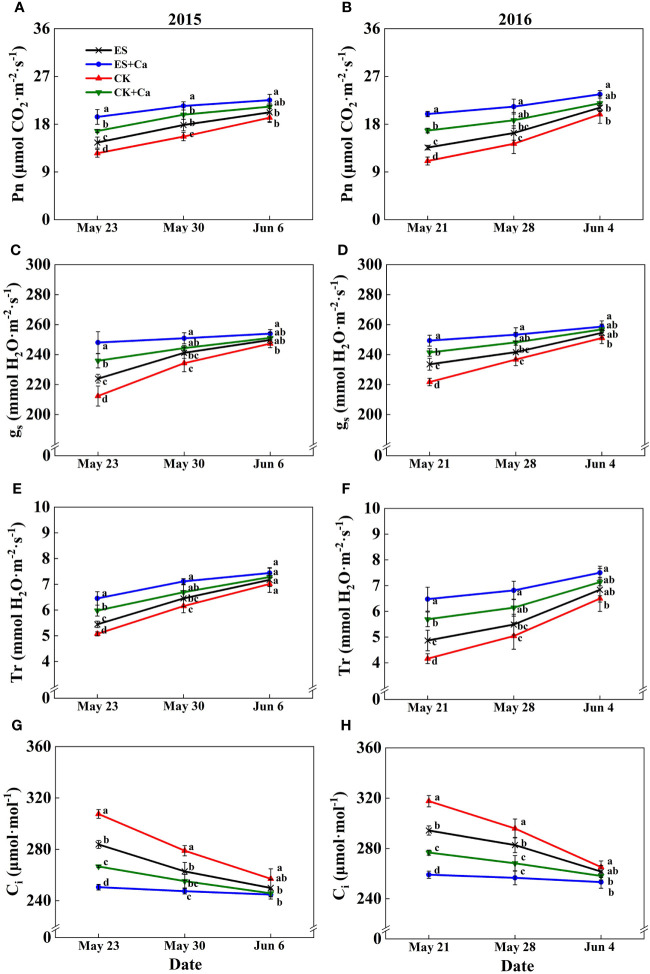
Effects of exogenous foliar calcium (Ca^2+^) application on peanut net photosynthetic rate (Pn, **A**, **B**), stomatal conductance (g_s_, **C**, **D**), transpiration rate (Tr, **E**, **F**), intercellular CO_2_ concentration (Ci, **G**, **H**) under early and normal sowing scenarios. Values are means ± SD (n = 3). Different letters indicate significant differences according to Duncan’s multiple range tests (*p* < 0.05).

**Figure 6:**
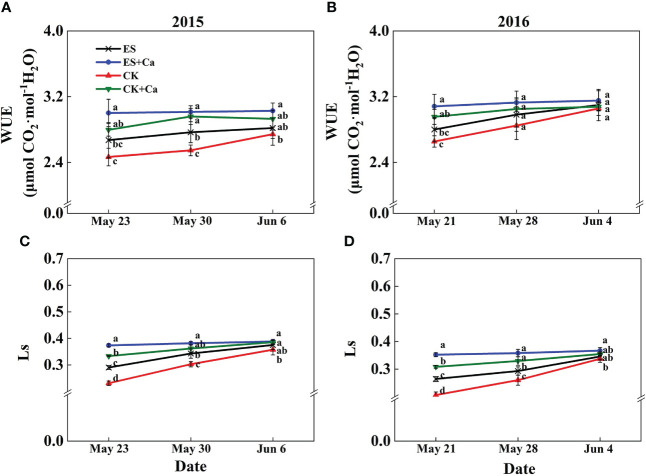
Effects of exogenous foliar calcium (Ca^2+^) application on peanut water-use efficiency (WUE, **A**, **B**) and leaf stomatal limitation (Ls, **C**, **D**) under early and normal sowing scenarios. Values are means ± SD (n = 3). Different letters indicate significant differences according to Duncan’s multiple range tests (*p* < 0.05).

The ES treatment increased Pn, gs, Tr, Ls, and WUE and decreased Ci when compared to CK. While ES+Ca and CK+Ca treatments further increased Pn, gs, Tr, Ls, and WUE and decreased Ci compared with that of ES. Moreover, as the peanut grows, the gas exchange characteristics did not differ between CK and ES treatments on Jun. 6, 2015, and Jun. 4, 2016, but they were still at a lower level than ES+Ca and CK+Ca treatments (**Figure 5**, **6**).

### 3.4 Effects of exogenous foliar calcium (Ca^2+^) application on peanut photosystem activities under early and normal sowing scenarios

**Figure 7:**
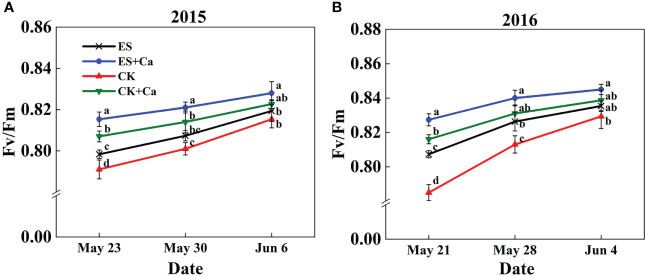
Effects of exogenous foliar calcium (Ca^2+^) application on peanut maximum quantum yield of PSII (Fv/Fm) under early and normal sowing scenarios during the **(A)** 2015 and **(B)** 2016. Values are means ± SD (n = 3). Different letters indicate significant differences according to Duncan’s multiple range tests (*p* < 0.05).

The Fv/Fm increased markedly under the ES treatment than CK. Furthermore, ES+Ca and CK+Ca treatments also enhanced Fv/Fm compared with the ES treatment. The Fv/Fm had no significant difference between ES and CK treatments on Jun. 6, 2015, and Jun. 4, 2016, but they were still lower than ES+Ca and CK+Ca treatments (**Figure 7**).

**Figure 8:**
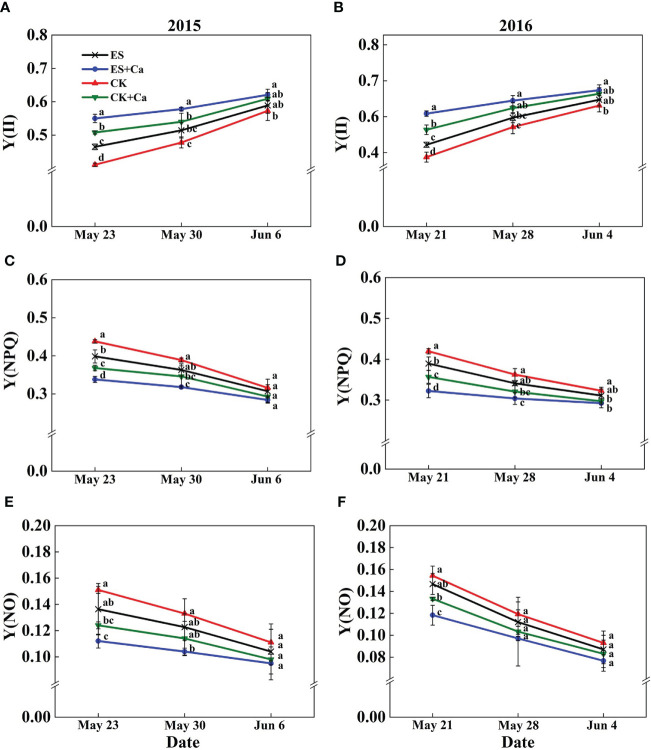
Effects of exogenous foliar calcium (Ca^2+^) application on peanut PSII photochemistry effective quantum yield [Y(II), **A**, **B**], PSII regulated energy dissipation quantum yield [Y(NPQ), **C**, **D**], PSII non-regulated energy dissipation quantum yield [Y(NO), **E**, **F**] under early and normal sowing scenarios. Values are means ± SD (n = 3). Different letters indicate significant differences according to Duncan’s multiple range tests (*p* < 0.05).

The ES treatment significantly enhanced Y(II) compared with that of CK. In addition, Y(II) in CK decreased markedly and dissipated excess energy by increasing the regulatory quantum yield of PSII [Y(NPQ)]. The dissipated excess energy depended mainly upon Y(NPQ) which was likely to be insufficient thus increasing Y(NO) to a higher level. Conversely, ES+Ca and CK+Ca treatments increased Y(II) and decreased Y(NPQ) and Y(NO). As the peanut growing season progresses, the Y(NO) did not differ between all treatments on Jun. 6, 2015, and Jun. 4, 2016 (**Figure 8**).

**Figure 9:**
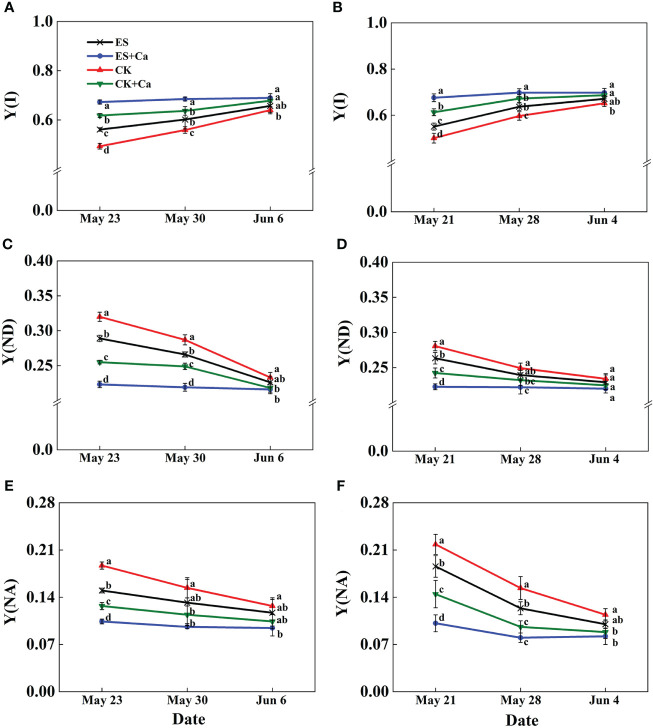
Effects of exogenous foliar calcium (Ca^2+^) application on peanut PSI photochemistry effective quantum yield [Y(I), **A**, **B**], PSI non-photochemical energy dissipation due to the donor-side limitation [Y(ND), **C**, **D**], PSI non-photochemical energy dissipation due to the acceptor-side limitation [Y(NA), **E**, **F**] under early and normal sowing scenarios. Values are means ± SD (n = 3). Different letters indicate significant differences according to Duncan’s multiple range tests (*p* < 0.05).

The ES treatment enhanced Y(I) and reduced Y(ND) and Y(NA) when compared to CK. Moreover, ES+Ca and CK+Ca treatments delivered higher Y(I) and lower Y(ND), and Y(NA) than the ES treatment. While Y(I), Y(ND), and Y(NA) had no significant difference between CK and ES treatments on Jun. 6, 2015, and Jun. 4, 2016, and they remained at a lower level than ES+Ca and CK+Ca treatments (**Figure 9**).

**Figure 10:**
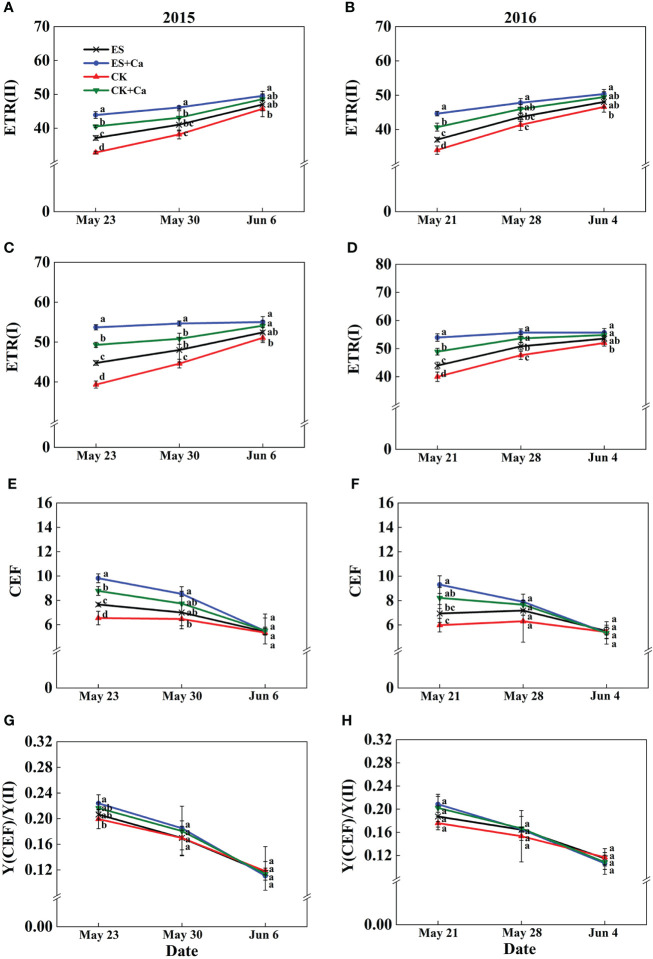
Effects of exogenous foliar calcium (Ca^2+^) application on peanut PSII photosynthetic electron transport rate [ETR(II), **A**, **B**], PSI photosynthetic electron transport rate [ETR(I), **C**, **D**], cyclic electron flow around PSI (CEF, **E**, **F**), the ratio of quantum yield of CEF to Y(II) [Y(CEF)/Y(II), **G**, **H**] under early and normal sowing scenarios. Values are means ± SD (n = 3). Different letters indicate significant differences according to Duncan’s multiple range tests (*p* < 0.05).

The ES treatment increased ETR(II), ETR(I), and CEF when compared with that of CK. Meanwhile, ES+Ca and CK+Ca treatments produced higher ETR(II), ETR(I), CEF, and Y(CEF)/Y(II) than the ES treatment. The CEF and Y(CEF)/Y(II) did not differ between all treatments on Jun. 6, 2015, and Jun. 4, 2016 (**Figure 10**).

### 3.5 Effects of exogenous foliar calcium (Ca^2+^) application on peanut yield under early and normal sowing scenarios

The ES treatment dramatically enhanced the number of full pods per plant and the 100-pod weight compared with that of CK. Meanwhile, the ES+Ca treatment further increased the number of full pods per plant and the 100-pod weight on different harvest dates (Sep. 25 and Oct. 9, 2015, Sep. 23 and Oct 7, 2016) compared with the CK and ES treatments (**Table 1**).

**Table 1:** Effects of exogenous foliar calcium (Ca^2+^) application on the number of full pods per peanut plant and the 100-pod weight under early and normal sowing scenarios in 2015 and 2016.

Years	Treatments	No. of full pods per plant (g)		100-pod weight (g)	
		**Sep. 25**	**Oct. 9**	**Sep. 25**	**Oct. 9**
2015	CK	15.7±0.6 c	18.0±1.0 c b	165.8±4.6 c	180.8±4.6 c b
	CK+Ca	18.0±1.0 b	20.3±0.6 b a	177.3±3.1 b	197.3±2.7 b a
	ES	18.0±1.0 b b	20.3±0.6 b	176.9±3.5 b b	193.5±8.2 b
	ES+Ca	20.7±0.6 a a	22.3±0.6 a	193.9±2.3 a a	209.1±2.9 a
		**Sep. 23**	**Oct. 7**	**Sep. 23**	**Oct. 7**
2016	CK	16.0±1.0 c	19.0±1.0 c b	172.3±4.1 c	183.5±2.6 c b
	CK+Ca	18.3±0.6 b	21.7±0.6 b a	183.3±3.9 b	203.7±5.7 b a
	ES	18.7±0.6 b b	21.3±0.6 b	183.4±3.4 b b	200.7±8.9 b
	ES+Ca	21.3±0.6 a a	23.3±0.6 a	200.0±3.8 a a	216.7±3.2 a

Values are means ± SD (n = 3). Different black letters (a, b and c) in the same column indicate significant differences according to Duncan’s multiple range tests (p < 0.05). Different red letters (a and b) in the same index indicate significant (p < 0.05) differences between CK (Oct. harvest date), CK+Ca (Oct. harvest date), ES (Sep. harvest date), and ES+Ca (Sep. harvest date) at the relative full maturity stages (Shown in red dashed box).

*Values are means ± SD (n = 3). Different black letters (a, b and c) in the same column indicate significant differences according to Duncan’s multiple range tests (p < 0.05). Different red letters (a and b) in the same index indicate significant (p < 0.05) differences between CK (Oct. harvest date), CK+Ca (Oct. harvest date), ES (Sep. harvest date), and ES+Ca (Sep. harvest date) at the relative full maturity stages (Shown in red dashed box).*

**Figure 11:**
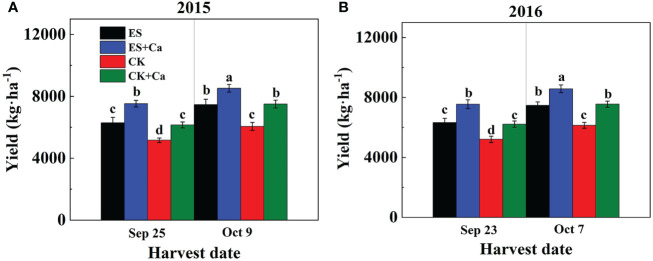
Effects of exogenous foliar calcium (Ca^2+^) application on peanut yield under early and normal sowing scenarios during **(A)** 2015 and **(B)** 2016. Values are means ± SD (n = 3). Different letters indicate significant differences according to Duncan’s multiple range tests (*p* < 0.05).

The ES treatment significantly increased peanut yield when compared to CK. Moreover, ES+Ca and CK+Ca treatments further increased peanut yield on different harvest dates. Compared to the CK, the ES treatment increased peanut yield by 21.9% and 21.4% in the Sep. harvest date in 2015 and 2016, respectively. While in the Oct. harvest date, the ES treatment increased peanut yield by 23.3% and 21.7% in 2015 and 2016, respectively, compared with that of CK. Compared to CK+Ca, the ES+Ca treatment increased peanut yield by 22.4% and 21.3% in the Sep. harvest date in 2015 and 2016, respectively. While the ES+Ca treatment increased peanut yield by 13.7% and 13.6% in the Oct. harvest date in 2015 and 2016, respectively, compared to CK+Ca. When plants were harvested at full maturity (ES and ES+Ca harvested in Sep., CK and CK+Ca harvested in Oct.), it was observed that peanut yield did not differ between ES and CK or ES+Ca and CK+Ca (**Figure 11**).

## 4 Discussion

### 4.1 Exogenous Ca^2+^ enhanced peanut growth and photosynthetic capacity under early sowing scenario in the field

Variations in temperature and precipitation were the primary factors that influenced the peanut seedling stage under the 15 d early sowing (daily minimum temperature [T_min_] > 13.4°C) and normal sowing (daily minimum temperature [T_min_] > 12.0°C) conditions (**Figure 1**). The chilling injury and other combined chilling injuries are the primary factors curtailing peanut production during the seedling vegetative stage (Allen and Ort, 2001; Zhou et al., 2017; Tian et al., 2019). Exogenous foliar Ca^2+^ application restored the inhibition of plant growth and biomass accumulation under the ES scenario (**Figures 2**, **3**), which was consistent with earlier research reported in maize and soybean (Sicher, 2011; Alsajri et al., 2019; Román et al., 2021). Unlike the regular sowing practice, the ES approach is likely to create new biological challenges such as changes in the duration of the nocturnal phase, and also the light-to-dark conversion chilling adaptations (Virk et al., 2020; Wu et al., 2020). Previous studies have shown that high levels of non-structural carbohydrates are useful in conferring cellular protection; these serve as either signaling substance(s) or act as osmoprotectants during cold acclimation (Sicher, 2011). Chilling stress not only damaged the chloroplast grana and expansion of starch grains but also inhibited nonstructural carbohydrate translocation from source to sink (Song et al., 2020; Wu et al., 2020; Liu et al., 2022). No matter whether in short-term or long-term chilling stress (including early sowing and normal sowing of early spring season), excessive accumulation of non-structural carbohydrates at the seedling stage would significantly lead to a negative feedback inhibition of leaves photosynthesis and causes an over-reduction of the photosynthetic electron chain to impair photosynthetic machinery and chloroplast morphology (Adams et al., 2013; Liu, 2020; Wu et al., 2020). Exogenous Ca^2+^ effectively promoted the export of nonstructural carbohydrates and then decreased the excessive accumulation of nonstructural carbohydrates in peanut photosynthesizing leaves under ES stress (**Figure 4**). Carbohydrate synthesis (such as soluble sugars, starch) occurs generally in mature leaf (sources) and follows a circadian rhythm (e.g., for sucrose) (Tovignan et al., 2016). The source-to-sink sugar translocation linked to photosynthesis is closely associated with plant growth (Brauer et al., 1990; Sonoike, 1996; Tan et al., 2011). Exogenous foliar Ca^2+^ application ameliorated photoinhibition due to the stomatal limitations in peanut photosynthesizing leaves under ES (**Figure 5**), which was consistent with findings in *Commelina communis* L (Suorsa, 2015). Peanut is a typical calciphilous plant and is vulnerable to photoinhibition caused by the accumulation of photosynthetic end-products (e.g. starch) (Virk et al., 2020; Wu et al., 2020). To maintain optimal growth, there must be sufficient sinks to utilize the translocated carbohydrates produced earlier by the sources through photosynthesis; thus, any disruption to these processes by unfavorable factors (e.g. chilling) will alter the source-sink relationship. Ca^2+^ regulates carbon assimilation by mediating the activity of deinoheptanose-1,7-diphosphatase (SBPase) and fructose-1,6-diphosphatase (FBPase) (two key enzymes in the Calvin cycle), where Ca^2+^ improved the synthesis, phloem loading, and export of photosynthetic carbohydrates (Brauer et al., 1990; Tan et al., 2011; Liu et al., 2013; Wu et al., 2020; Liu et al., 2022). Thus, exogenous Ca^2+^ alleviated the early sowing chilling-dependent feedback limitation on peanut photosynthesis by increasing growth/sink demand.

### 4.2 Exogenous Ca^2+^ alleviated the adverse photodamage of photosystems under the early sowing scenario in the field

Exogenous Ca^2+^ alleviated the adverse photodamage of PSII and PSI, which promoted photosynthetic electron transport flow under early and normal sowing scenarios (**Figures 7**–**10**). Exposing the leaves of *Calathea makoyana* to chilling in darkness did not affect photosystems’ activities and visual appearance, but chilling in the light led to severe photodamage and leaf necrosis (Hogewoning and Harbinson, 2007). Both PSI and PSII are known to be sensitive to excess light under chilling stress; PSII is easily inactivated by an excess of excitations and PSI is more prone to potential photo-damage caused by excess electrons arriving from PSII and the decrease in the rate of photosynthetic carbon fixation (Wang et al., 2016; Huang et al., 2016). Chilling in light will decrease the production of △pH with the accumulation of NADPH and reduce the production of ATP, which inhibited CO_2_ fixation (Yamori and Shikanai, 2016; Huang et al., 2021). The stress conditions will start photosystem protective mechanism, such as stimulation of violaxanthin de-epoxidase (VDE) activity and increase the de-epoxidation state of xanthophyll pigments to accelerate NPQ, or activate the cyclic electron flow (Ding et al., 2017; Paredes and Quiles, 2017; Ma et al., 2021). Exogenous calcium priming is necessary when environmental conditions are extremely harsh and self-protection mechanisms are not enough to resist environmental stress (Liu, 2020). Ca^2+^ can bind to its receptor protein CaM and produce a Ca^2+^-CaM complex by activating phosphodiesterase, Ca^2+^-ATPase, and decarboxylase at low temperatures (Ranty et al., 2006; Li et al., 2017). Meanwhile, exogenous Ca^2+^ (i) promoted the photosystem self-repairing by enhancing the calcium-binding protein (Terashima et al., 2012); (ii) increased △pH which promoted Ca^2+^ combined with PsbO to stabilized PSII reaction center (Yi et al., 2005); (iii) reduced the accumulation of NADPH (Zhang et al., 2014); (iv) enhanced ATPase activity and CEF, which effectively ameliorated the PSII and PSI-linked photochemical disruptions (**Figure 10**, Song et al., 2020). In addition, cold signal-induced calcium may trigger downstream signaling molecules such as CaM, ROS, and Rbohs-respiratory burst oxidase (= NADPH oxidase) fully regulated in the photoreaction and carbon assimilation of chloroplasts (Pirayesh et al., 2021). In particular, exogenous Ca^2+^ could promote endogenous [Ca^2+^]_cyt_ entering thylakoid lumen regulated by a light signal, and enhanced intracellular [Ca^2+^]_cyt_, which can maintain oxygen release complex (OEC) activity and equilibrium regulation of transmembrane proton dynamics (Krieger and Weis, 1993; Liang et al., 2009; Huang et al., 2016).

### 4.3 Exogenous Ca^2+^ enhanced peanut yield under the early sowing scenario in the field

In general, ES effectively avoids drought stress and improved peanut yield significantly (Wan, 2003; Zhang et al., 2022). When ES was combined with exogenous foliar Ca^2+^ application, the stimulation effect on yield will be amplified significantly (**Table 1**). Previous studies showed that crop yield tends to decrease with later sowing (Sallam et al., 2017; Manning et al., 2020). Early sowing improves water availability (Soriano et al., 2004) and delivers a harvest rapidly for early high-price markets or extends the growth duration (Zhou et al., 2018). In our study, exogenous Ca^2+^ priming enhanced the peanut pod yield under early and normal sowing scenarios (**Table 1**). Compared to the CK, the ES treatment significantly increased peanut yield by 21.9% and 21.4% in the Sep. harvest date in 2015 and 2016, respectively. While in the Oct. harvest date, the ES treatment increased peanut yield significantly by 23.3% and 21.7% in 2015 and 2016, respectively. In particular, ES+Ca treatment further increased peanut yield significantly by 13.7% and 13.6% in the Oct. harvest date in 2015 and 2016, respectively (**Figure 11**). It was indicated that exogenous Ca^2+^ significantly improved plant growth, leaf extension, the accumulation of photosynthates and pod yield in peanut seedlings, which is consistent with early studies (White and Broadley, 2003; Hepler, 2005; Zhang et al., 2020; Li et al., 2022; Liu et al., 2022). During early spring, especially in 40°N high latitude areas, an increase in yield and the concomitant economic value can be obtained by implementing proper early sowing cultivation strategies (Román et al., 2021). Thus, exogenous Ca^2+^ applications could further increase peanut yield under any ES and NS scenarios and offer practical options for growers to improve the yield through various early and late cultivation practices in a high-altitude area.

## 5 Conclusions

Exogenous Ca^2+^ applications were proven effective in restoring peanut photosynthesis and pod yield during early sowing and normal sowing scenarios in northern China. The two years experiments demonstrated that exogenous Ca^2+^ promoted foliar and root growth, alleviated PSI and PSII photoinhibition, and ameliorated the ES-linked photosynthetic feedback inhibition. Specifically for peanuts, moderate (15 mM) exogenous foliar Ca^2+^ application could improve the pod yield at both early and late harvest dates and include scenarios where cold-stress periods are possible. From the peanut growers’ perspective, the simple Ca^2+^ priming approach provided an effective biotechnological solution and delivered a concomitant financial guarantee to secure peanut production against potential abiotic stress periods commonly encountered in high latitudes.

## Data availability statement

The original contributions presented in the study are included in the article/**Supplementary Material**. Further inquiries can be directed to the corresponding author.

## Author contributions

YL, QBS, SZ, and CB designed the experiment. QBS, CB, and DW conducted the study and collected data for preliminary analysis. YL, QWS, XH, TL, and JY further analyzed the data and prepared the manuscript. All authors reviewed and commented on the manuscript. All authors contributed to the article and approved the submitted version.

## Funding

This research was funded by the National Natural Science Foundation of China (project nos. 31772391 and 31301842), ARC-Linkage Project (project no. LP200100341), and the Scientific Research Project of the Education Department of Liaoning Province, China (project no. LJKZ0661).

## Acknowledgments

We sincerely thank the reviewers for all their valuable comments and recommendations to improve the manuscript.

## Conflict of interest

The authors declare that the research was conducted in the absence of any commercial or financial relationships that could be construed as a potential conflict of interest.

## Publisher’s note

All claims expressed in this article are solely those of the authors and do not necessarily represent those of their affiliated organizations, or those of the publisher, the editors and the reviewers. Any product that may be evaluated in this article, or claim that may be made by its manufacturer, is not guaranteed or endorsed by the publisher.

## Glossary

**Table:**

ATP	Adenosine triphosphate
Ca	Atmospheric CO2 concentration
Ci	Intercellular CO2 concentration
CEF	Cyclic electron flow
ES	Early sowing chilling
ETR(I)	Relative electron transport rate in Photosystem I
ETR(II)	Relative electron transport rate in Photosystem II
F	Fluorescence yield measured briefly before application of a saturation pulse
Fo	Minimal fluorescence yield of the dark-adapted sample with all PSII centers open
Fo’	Minimal fluorescence yield of the illuminated sample with all PSII centers open
Fm	Maximal fluorescence yield of the dark-adapted sample with all PSII centers closed
Fm’	Maximal fluorescence yield of the illuminated sample with all PSII centers closed
Fv/Fm	Maximal photochemistry efficiency in Photosystem II
FH1	Fenghua 1
gs	Stomatal conductance
LA	Leaf area
Ls	Leaf stomatal limitation
NAD	Nicotinamide adenine dinucleotide
NADK	Nicotinamide adenine dinucleotide kinase
NADP	Oxidation form of nicotinamide adenine dinucleotide phosphate
NADPH	Nicotinamide adenine dinucleotide phosphate
NPQ	Nonphotochemical quenching
PAR	Photosynthetically active radiation measured in mmol quanta m–2 s–1
Pm	Maximal P700 signal
Pm’	Realtime
P700 signal under light	
Pn	Net photosynthetic rate
PPFD	Photosynthetic photon flux density
Pred	P700 reduction coefficient under light
PSI	Photosystem I
PSII	Photosystem II
RH	Relative humidity
Tr	Transpiration rate
WUE	Water-use efficiency
Y(II) = FPSII	Actual quantum yield in PSII under light
Y(NO)	Non-regulatory quantum yield in PSII under light
Y(NPQ)	Regulatory quantum yield in PSII under light
Y(I) = FPSI	Actual quantum yield in PSI under light
Y(ND)	Quantum yield of PSI non-photochemical energy dissipation due to donor-side limitation
Y(NA)	Quantum yield of PSI non-photochemical energy dissipation due to acceptor-side limitation
